# On Some Points in the Surgery of Hernia

**Published:** 1856-03

**Authors:** Nathaniel Ward

**Affiliations:** Assistant Surgeon to the London Hospital


*On some points in the Surgery of Hernia.* By NATHANIEL WARD, ESQ., F. R. C. S., Assistant Surgeon to the London Hospital.

Whenever a patient is seized with pain in the umbilical region, accompanied with nausea or vomiting, the propriety of examining the abdominal apertures should be regarded as a cardinal rule. This rule, however, in consequence of the oomparative rarity of rupture in private practice, is, I fear, apt to be somewhat slighted; and it now and then happens that a case is treated for many hours as one of dyspepsia, colic, or enteritis; leeches, counter-irritation, cataplasms, fomentations and drugs are ineffectually brought to bear, followed by an aggravation, instead of a diminution of the symptoms. An examination of the abdominal walls is subsequently instituted, and a hernial tumor probably detected. An operation is performed, and the chances of death will mainly depend on the time it has been deferred. I am personally acquainted with one or two cases of such fatal oversight. The ignorance and false modesty of patients frequently also aids the surgeon in the establishment of a faulty diagnosis, and it will be found, strange as it may appear, that patients suffering from strangulated hernia now and then are quite unconscious of the existence of any swelling, or if they know of it, are surprised on being informed that the severe symptoms under which they are suffering have any thing to do with the swelling they have had for years, particularly as the pain that has accompanied these symptoms is usually referred to a part of the abdomen remote from the position of the tumor. My friend, Mr. Ray, of Sittingbourne, informs me that on one occasion an elderly female was operated on by him, who, he found on inquiry, had been suffering from symptoms of strangulation for thirteen days. During this period she had consulted no one, nor would her husband let her, but relied on his own medical powers, which consisted in the use of some simple medicines, and a recommendation to take exercise for her relief, so that the patient had accomplished several long walks during this interval. On Mr. Ray being eventually called to see her, and on his explanation of the nature of her affection, the astonishment and incredulity depicted on the countenance of the wife and husband was great, and the former remarked, before consenting to an operation, "Now, doctor dear, do you really think that this here (putting her hand on the groin) has any thing to do with the sickness?" The poor creature, I need hardly say, died, as on opening the sac a false anus became soon established, and she sank rapidly. When a tumor has been detected in, or protruding from, say one femoral, or one inguinal canal, the examination should not even then be deemed sufficient, but all the abdominal apertures should be examined. However redundant such precaution may appear in the majority of cases, experience forcibly dictates the necessity of attending to it, and the mere circumstance of one fatal case having taken place from such deficiency of supervision, fully warrants its adoption. A large, fat woman was admitted, suffering from symptoms of strangulation of more than usual severity. Examination detected a large umbilical hernia. This was reduced without much difficulty, but the symptoms were not relieved. It came down again, and then could not be returned, and an operation was performed. Omentum only was found in the rupture. The symptoms of strangulation continued, and she died. On a post-mortem examination a knuckle of intestine was found in the femoral ring, perfectly sphacelated. There had been also considerable peritonitis.

Independently of a small hernia being overlooked, when the attention is concentrated on a large one somewhere else, two or three herniæ may be detected at the same time, and there being severe strangulation symptoms, it becomes a question, which of the protrusions, or whether more than one, should be selected for the operative proceedings of the surgeon. The late Mr. Robinson, in a valuable paper, "On the Complications of Hernia," which was published in the *London Journal of Medicine*, quotes the following case: "Mr. Luke was called to a female who was laboring under an inguinal and an umbilical hernia, with symptoms of strangulation. Both ruptures were very tense and painful, and both were irreducible. The former was small, and was apparently contained in the inguinal canal; of the two it was the more tense, and could be obscurely felt through a thick layer of fat." He was induced to operate on this one. He found, upon opening the sac, a portion of small intestine tightly strangulated. He divided the stricture, and returned the bowel. Relief followed; the symptoms subsided, and the woman recovered.—*London Lancet.*

